# A Cone Bean Computer Tomography Investigation of the Newly Formed Mandibular Anterior Ridge following the Treatment of an Extended Comminuted Fracture: A 12-Year Follow-Up

**DOI:** 10.1155/2024/1824016

**Published:** 2024-02-21

**Authors:** Pascal Grün, Florian Pfaffeneder-Mantai, Patrick Bandura, Benedikt Schneider, Anna Sophia Bandura, Dritan Turhani

**Affiliations:** ^1^Center for Oral and Maxillofacial Surgery, Department of Dentistry, Faculty of Medicine and Dentistry, Danube Private University, Steiner Landstraße 124, 3500 Krems, Austria; ^2^Division for Chemistry and Physics of Materials, Department of Medicine, Faculty of Medicine and Dentistry, Danube Private University, Steiner Landstraße 124, 3500 Krems, Austria

## Abstract

**Introduction:**

Extensive comminuted fractures are associated with tooth loss that ultimately leads to dimensional changes in the hard and soft tissues of the alveolar ridge. Reconstruction of the lost mandibular anterior ridge is very complex due to the natural curvature of the region. *Case Presentation*. In this case report, the combination of the modified shell technique with autologous bone plates and the guided bone regeneration (GBR) technique was performed on an 18-year-old patient after a comminuted fracture, to ensure new bone formation in the anterior ridge with a natural curvature. After the treatment progressed without complications, three dental implants were placed. Annual cone beam computed tomography (CBCT) images were obtained and evaluated using the GNU Image Manipulation Program (GIMP© 2.10). This allowed measurements of the buccal and lingual bone around the implants, showing the annual bone loss in a twelve-year observation period. *Discussion*. The treatment of the comminuted fracture and the combination of the modified shell technique with autologous bone plates, the GBR technique, and implant placement can be considered successful. The three dental implants were osseointegrated in 2010, with the buccal bone level averaging 1.31 mm below the implant shoulder and the lingual bone level 1.57 mm above the implant shoulder. In 2021, the measurements showed a bone loss of 0.99 mm at the buccal implant shoulder and 0.69 mm at the lingual implant shoulder.

**Conclusion:**

The combination of the modified shell technique with autologous bone plates and the GBR technique is a reliable method to ensure new bone formation in the anterior ridge. The use of CBCT is an excellent method to evaluate bone resorption around dental implants, but due to minimal bone resorption in the observation period, an annual CBCT examination is exaggerated.

## 1. Introduction

Mandibular fractures are a common injury occurring due to trauma to the face and jaw, and a mandibular fracture itself may be closed, open, comminuted, or displaced [1].

A comminuted fracture is defined by the presence of multiple fracture lines resulting in many small pieces within the same area of the mandible (e.g., angle, body, ramus, or symphysis) [2]. Regarding extensive comminuted fractures, multiple sites of the mandible are fragmented, crushed, pulverised, or broken into multiple pieces [3]. The most common causes of comminuted fractures are traffic accidents (40-42%), falls from great heights, sports accidents, or work accidents [4]. Traditionally, extensive comminuted fractures of the mandible were considered an indication for closed reduction to avoid periosteal stripping and devascularization of the comminuted segments [5]. However, in recent years, the treatment perspective for such injuries has changed because of advances in rigid fixation techniques. The literature suggests that open reduction and internal fixation are better treatment options for some mandibular comminuted fractures and have a lower complication rate than closed reduction. Yet, it remains controversial whether a closed reduction is the optimal treatment for comminuted fractures compared with open reduction and internal fixation [6].

Further, comminuted fractures are associated with tooth loss, triggering a sequence of biological events that ultimately lead to dimensional changes in the hard and soft tissues of the alveolar ridge [7]. Some degree of alveolar ridge resorption is unavoidable after tooth loss [8]. The loss of the vertical ridge height on the mesial and distal sides of an extraction socket is particularly severe [9]. Depending on the severity, a comminuted mandibular fracture can result in complications such as dysfunction of the inferior alveolar nerve and postoperative complications such as bone loss in general, the loss of teeth, or the loss of the alveolar ridge [10].

Alveolar ridge reconstruction in combination with the placement of dental implants is essential to achieve a long-term functional outcome for prosthetic restorations in patients after an extended comminuted fracture treatment [11].

There are two main options to increase bone volume prior to implant placement: additive and expansive methods. In additive methods, the bony defect is reconstructed in height and width with an onlay of augmentation material. These may include bone block grafts [12], guided bone regeneration (GBR) techniques [13], or augmentation with titanium meshes [14]. Expansive techniques such as bone splitting [15] or distraction osteogenesis result in the widening or elevation of the alveolar ridge [16]. Autologous bone grafts are still considered the gold standard of augmentation techniques [17]. Several studies show that these grafts are highly suitable for increasing bone volume prior to implant placement [18]. Nevertheless, the main disadvantage is that a donor site is required. Various complications may occur at these donor regions, such as changes in pulpal sensitivity, superficial skin sensory disturbances, and postoperative pain during mastication [19].

**Figure 1:**
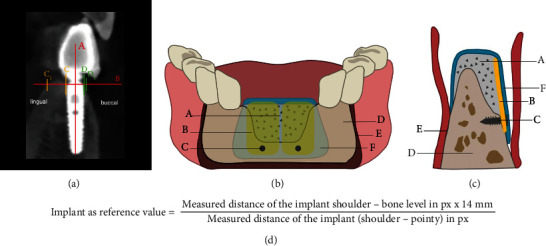
(a) CBCT: an example diagram showing how the measurements were taken ((A) implant length for reference (manufacturer information); (B) shoulder of the implant; (C, C^1^) distance for lingual measurements were recorded from the implant shoulder (C) to the bone level (C^1^); (D, D^1^) distance for buccal measurements were recorded from the implant shoulder (D) to the bone level (D^1^). (b) Graphical representation (frontal section): the combination of the modified shell technique with autologous bone plates and the guided bone regeneration technique ((A) mixture of milled bone chips and bone substitute; (B) bone plates; (C) osteosynthesis screws; (D) alveolar ridge; (E) periosteum; (F) collagen membrane). (c) Graphical representation (sagittal section): combination of the modified shell technique with autogenous bone plates and the guided bone regeneration technique. (d) The formula given for determining the implant length served as a reference for converting the measured pixels into millimetres.

To combine the advantages of the low resorption rate of cortical bone and the osteoconductive properties of cancellous bone, the “three-dimensional” reconstruction or “shell technique” was developed. During this procedure, a thin cortical bone plate is fixed at a distance from the alveolar ridge with osteosynthetic screws [20]. The gap between the ridge and the graft is then filled with either autogenous bone particles [21] or with a mixture of bone substitute material and autologous bone [22]. A modification of the shell technique using a combination of autogenous bone block grafting and guided bone regeneration (GBR) can reduce resorption processes of autogenous monocortical bone blocks (Figures 1(b) and 1(c)) [17, 23, 24]. This method of “augmentative relining” allows for an increase in bone volume at the augmentation site and aids in the incorporation of the bone substitute granules into the regenerated bone [24].

The aim of this case report was to track the results of the modified shell technique around the subsequently placed dental implants in a newly built mandibular alveolar ridge after the treatment of an extensive comminuted fracture. This was achieved by using a cone beam computed tomography (CBCT) to evaluate the amount of bone gain over a 12-year follow-up period. The modified shell technique prior to the insertion of the dental implants was performed using a retromolar bone plate, a mixture of particulate bovine xenograft and autologous bone particles, covered with a collagen membrane.

## 2. Case Presentation

The requirements of the Declaration of Helsinki were met, and the patient gave informed consent to all surgical procedures.

This case report documents a retrospective CBCT analysis of a surgical technique. The described modified shell technique is an additional well-described technique for horizontal augmentation procedures. Therefore, the case report was exempted from the approval process of the institutional review board.

**Figure 2:**
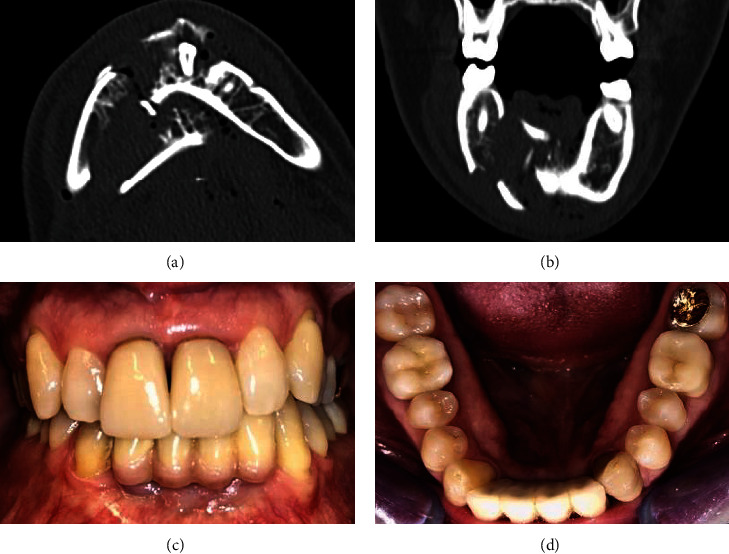
(a) CBCT horizontal view: facial skull with comminuted fracture in the mandible. (b) CBCT vertical view: facial skull with comminuted fracture in the mandible. (c) Buccal view of the clinical situation 12 years after comminuted fracture. (d) Lingual view of the clinical situation 12 years after comminuted fracture.

**Figure 3:**
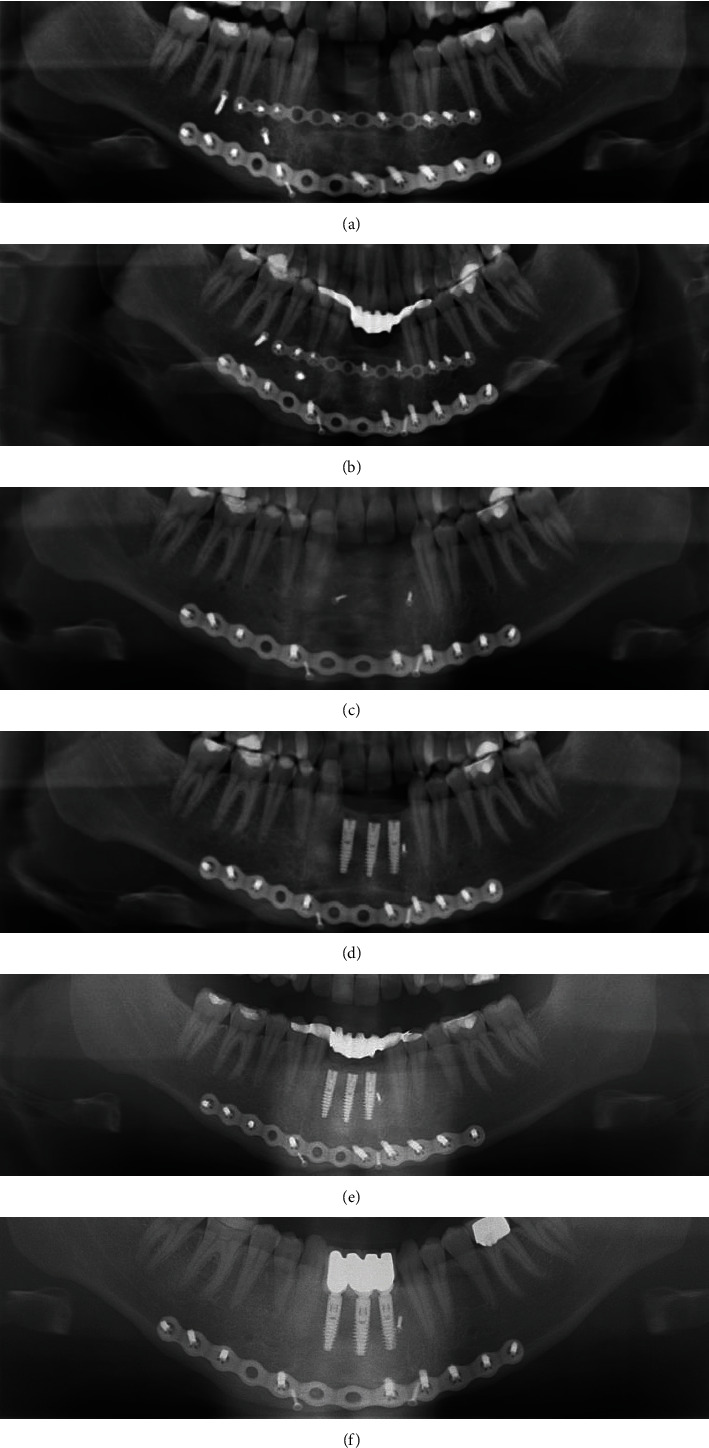
(a) OPT: postoperative treatment of the comminuted fracture with two osteosynthesis plates and two lag screws. (b) OPT: postoperative restoration of the switching gap with an adhesive bridge. (c) OPT: removal of an osteosynthesis plate at 10 months after the accident. (d) OPT: insertion of three Ankylos® implants with the dimensions 3.5 × 14 mm. (e) OPT: after inserting the three Ankylos® implants, the switching gap was closed again with an adhesive bridge. (f) OPT: definitive restoration of the implants with a metal-ceramic bridge in the anterior part of the mandible.

An 18-year-old male fell from a height and sustained a deep laceration wound to the chin compounded with an extensive comminuted mandibular fracture. Radiological examination shows a comminuted fracture in the mandible with clear dislocation at several levels, fractured teeth in the anterior region from region 33 to 43 (FDI tooth-numbering system) (Figures 2(a) and 2(b)), and dislocation of the lingual cortices in region 43-47 into the floor of the mouth. This not only resulted in instability of the entire mandibular corpus, but also in air entrapment throughout the floor of the mouth. Open reduction and internal fixation of the mandibular comminuted fracture were performed using two locking reconstruction plates and screws, under general anaesthesia. The locking reconstruction plates (Synthes® MatrixMANDIBLE™ Plating System, Oberdorf, Schweiz) were adjusted and fixed with screws. Marginal bone segments were reduced and fixed to the reconstruction plate with screws. Small or nonmarginal bone segments were reduced and fixed to each other. Soft tissue was attached to the periosteum and sutured with Propylene® 4.0 (Ethicon, Raritan, New Jersey, USA) (Figure 3(a)). The postoperative course was free of complications, and the edentulous space was closed with an adhesive bridge (Figure 3(b)).

Ten months after the accident, the patient was readmitted to the operation room under intubation anaesthesia, during which the two osteosynthesis plates were removed. Intraoperatively, it was noticed that the fracture line of the lower jaw was not fully healed to the lower edge of the mandible. Therefore, the lower plate had to remain in place (Figure 3(c)), and in addition, bone loss was observed in the vertical and horizontal dimensions, which was sought to be restored. Thus, the plan was to harvest a 2 × 1.5 cm bone block from the right retromolar region. The required bone volume for the patient was determined through preoperative CBCT analysis of the recipient site. The ramus was the chosen site due to the availability of the required cortical bone volume and reduced morbidity compared to the symphysis [25].

At the donor site, buccal and lingual infiltration with 5 ml Ultracain Dental Forte® (SEPTODONT GmbH, Niederkassel, Germany) were given near the last molar, and an inferior alveolar nerve block was not used to avoid masking potential signs of iatrogenic nerve injury during surgery [26].

Using a midcrestal incision in the region of the lower left first molar and second bicuspid, a sulcular incision was made along the left lower second molar and first bicuspid, with a relieving incision at the distal line angle of the left lower second molar to the ascending ramus. This relieving incision also gave access to the donor site for the bone graft. A full-thickness flap was elevated, and, for tension-free wound closure, the periosteum was slit basally to the flap at the very beginning of the surgery to prevent bleeding until the membrane was inserted at the end of the procedure. The bone defect was measured using a periodontal probe to determine its size. The bone shell as well as the cortical plate were perforated with a bur for better angiogenesis of the graft. In addition, the mesial and distal sides were adjusted for a precise fit of the shell.

The augmented bone was harvested from the left ascending ramus of the mandible. A corticocancellous bone block was obtained with the help of an ultrasonic knife (Piezosurgery®; Mectron, Cologne, Germany). The harvested retromolar bone block had a thickness of approximately 3 mm. Using a bone mill (Bull Bone Mill®; Mondeal, Mühlheim an der Donau, Germany), the block was decreased to a thickness of less than 1 mm and used as the shell. The milled bone chips were mixed with autogenous blood and used as a particulate bone for the augmentation.

The donor site was then prepared by elevating buccal and lingual mucoperiosteal flaps in the anterior mandibular region. On the lingual side, a Bio-Gide® membrane (Geistlich, Wolhusen, Switzerland) was inserted between the bone and the periosteum.

Next, the recipient site was prepared to receive the bone graft, and the previously harvested bone plates were adapted to the defect. One of the bone plates was rounded at the edges to avoid injury to the mucosa and then inserted buccally into the remaining mandibular bone and secured with two screws. Once the bone plates were firmly screwed in place, a mixture of milled bone chips and bone substitute material was packed between the plates and the membrane to improve the graft's shape and quality.

The bone graft was covered with an absorbable collagen membrane. Subsequently, the mucosa was mobilised to the extent that tension-free wound closure was possible. Both horizontal mattress and single-button sutures with Propylene® 4.0 were used in this area. Postoperative care included Augmentin® (1,000 mg; twice daily for 5 days), a combination of analgesics and nonsteroidal anti-inflammatory drugs (three times a day).

Wound healing showed no complications. The single sutures at the retromolar donor site and at the grafted site were removed on day 10, and the mattress sutures were removed on day 14 after the surgery. After 2 weeks, the wound was primarily closed without any signs of inflammation. The patient followed a strict oral hygiene protocol during the follow-up period.

After another six months, the patient was operated on once again, this time under local anaesthesia. When drilling for the three implants, the bone presented itself as an excellent implant site. The three Ankylos® implants (Dentsply Sirona, Bensheim, Germany) 3.5 × 14 mm could be placed without any problems as there was sufficient bone thickness in the working site. All implants were primarily stable, but in this case, closed healing was chosen to avoid an unnecessary risk for the augmentation (Figure 3(d)). Over the next six months, the patient continued to use the adhesive bridge (Figure 3(e)).

After these six months, the three implants were exposed, and the dentist performed a temporary loading of the implants for another six months. During this time, there were neither problems with any implant nor with the provisional crowns.

After an additional seven months, a fixed gold metallic-facing ceramic bridge was inserted in the front of the lower jaw (Figure 3(f)).

Follow-up and annual controls took place in 3 different centres. No pathological symptoms or patient satisfaction issues were identified even after 12 years (Figures 2(c) and 2(d)).

## 3. Materials and Methods

### 3.1. Case Report Design and Data Collection

The case report design and protocols used in this article were reviewed and approved. This case reports a retrospective 12-year follow-up of one male patient. A consent declaration for using intraoral and radiologic photos was obtained by the author. In 2009, three Ankylos® 3.5 × 14 mm implants were placed in the lower front jaw after successful augmentation. The 18-year-old patient received postoperative instructions, and an adhesive bridge was applied. Each of the 3 implants was examined using a cone beam computed tomography (CBCT). These CBCTs were made in several different clinics to evaluate any potential bone loss. An appropriate literature research was achieved by studying medical and dental records in electronic and paper form.

### 3.2. Radiographic Measurements

Images of the patient's mandibular arch were acquired with the CBCT i-CAT Model 17-19 (Imaging Sciences International LLC, Hatfield, Pa). The imaging parameters were set to 120 kVp, 18.66 mAs, a scan time of 20 seconds, and a resolution of 0.4 mm.

The change in bone level around the implants was thus measured annually from 2010 to 2021 with the CBCT radiographs. The same image sections were generated in the radiographs with the following objectives: to show details in the sagittal plane and to use the largest cross-section of the implants with the same angles. However, due to incompatible file formats, the image sections were exported from digital volume tomography to “JPEG.”

Accurate measurements of potential bone loss around the implants on digital radiography images are now possible using the GNU Image Manipulation Program (GIMP 2.10), which is freely available for the analysis of digital images [27].

GIMP contains several tools that are useful for the enhancement and investigation of features seen in panoramic images [27].

Among other factors, using GIMP© 2.10 offers the possibility to measure potential bone loss. GIMP© 2.10 also offers the possibility to measure distances in continuous pixels. The implant length was used as a reference (manufacturer information), and centric measurements were taken from the implant shoulder to the tip of the implant. Using an orthogonal auxiliary line, perpendicular measurements were recorded from the implant shoulder (C and D in Figure 1(a)) to the bone level on both sides: buccal (distance from D to D^1^ in Figure 1(a)) and lingual (distance from C to C^1^ in Figure 1(a)). Both lines were drawn at right angles to achieve comparable and replicable results. Since the implant length served as a reference, the measured pixels could be converted into millimetres (Figure 1(d)). All measurements were performed by the same calibrated examiner. For further analysis, five measurements were taken for each distance and entered into a Microsoft Excel spreadsheet. The average difference between the measurements was 0.15 mm.

## 4. Results

**Figure 4:**
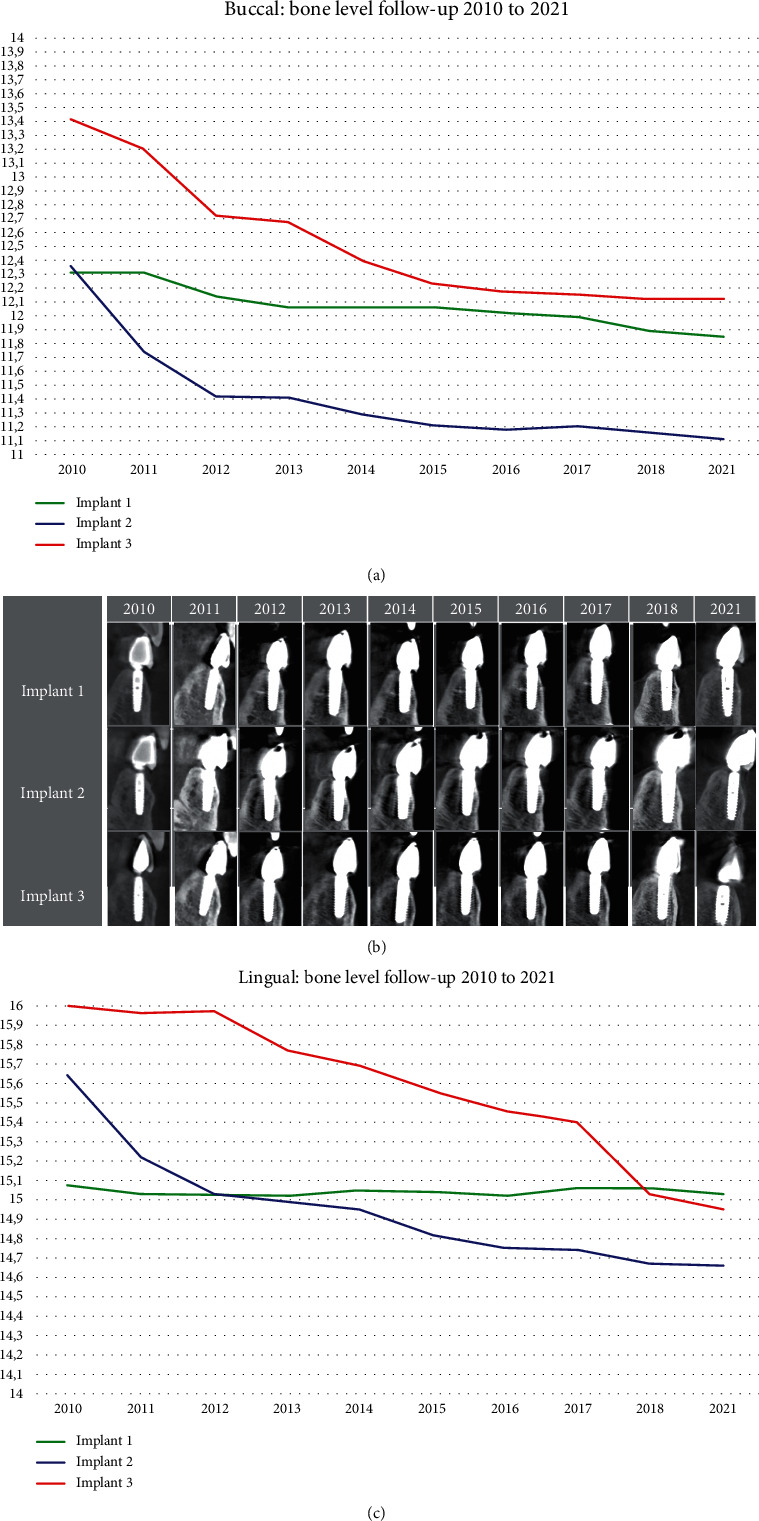
(a) A graph showing the buccal bone level from 2010 to 2021 in mm, with each line representing the bone level for each implant. (b) CBCT: analysed CBCT image from 2010 to 2021 of each implant (implant 1: region 32; implant 2: region 31/41; implant 3: region 42). (c) A graph showing the lingual bone level from 2010 to 2021 in mm, with each line representing the bone level for each implant.

The example picture shows how the measurements were conducted (Figure 1(a)), and the graph shows the buccal bone levels of the three implants in a twelve-year follow-up period (Figure 4(b)). The *x*-axis represents each year from 2010 to 2021. The *y*-axis displays the height of the bone level in mm. A range from 11 mm to 14 mm was chosen because the bone levels of all three implants were lower than the implant shoulder, which leads to the negative values. We concluded that the average buccal bone level for all implants decreased by 0.97 mm.

Similar to Figure 4(a), Figure 4(c) represents the lingual bone levels using the same method. However, the *y*-axis has a range from 14 mm to 16 mm because the bone levels were always higher than the implant shoulder.

In both graphs, the *y*-axis is relative to the implant length, in which 14 mm represents the top of the implant shoulder and, therefore, the top of the implant. The coloured lines in both figures (Figures 4(a) and 4(c)) illustrate the bone level of each implant as a progression through the years. Furthermore, the plus-minus signs (±) indicate the furthest collected value from the average, thus the uncertainty. The principle idea is to investigate an annual decrease in the bone level, but the results are, in some cases, so similar that the average of the respective measurements is sometimes higher than in previous years. There is an average lingual bone loss of 0.65 mm for all implants during this period.

We see that the implant in region 32 has the least amount of bone loss: lingual 0.01 mm and buccal 0.42 mm over the course of the complete follow-up. Both implants in region 31/41 and region 42 have nearly the same level of bone loss during the observation period—in region 31/41: lingual 0.97 mm and 1.19 mm buccal. In region 42, the average bone loss was 0.97 mm lingually and 1.29 mm buccally. Furthermore, buccally higher values are recorded than lingually for each implant.

Figure 4(a) displays every analysed CBCT image section. Upon radiographic evaluation, all implants showed sufficient mineralisation of the grafted autologous bone during the complete follow-up between 2010 and 2021.

## 5. Discussion

The treatment of mandibular comminuted fractures is challenging even for experienced surgeons. Accurate reduction and fixation of the fragments are challenging, especially when the anatomical references or occlusal relationships have been completely lost [28].

In addition, common postoperative complications such as inferior alveolar nerve dysfunction, general bone loss, tooth loss, or loss of the alveolar ridge may occur [10].

To preserve the vascular supply to the fragmented fragments and avoid secondary infection, closed reduction has long been considered the treatment of choice. However, recent reports have shown that open reduction and internal fixation (ORIF) are better treatment options with lower complication rates [6, 29]. Advances in surgical methods and the potential for more robust and reliable internal fixation have favoured ORIF in the treatment of mandibular comminuted fractures. It has also been suggested that closed reduction or conservative treatment is the better choice for minimally displaced comminuted fractures [5].

In our case, no complications were noted, and the alveolar ridge was successfully reconstructed using the modified shell technique and the GBR technique.

The use of these thin cortical bone blocks in preimplant surgery had several functions. The plate of cortical bone acted as a biocompatible membrane with high stiffness and was thus able to maintain the vertical dimension of the augmentation, providing a suitable atmosphere for bone regeneration.

Filling the vertical gap with a mixture of particulate bone and bone substitute material has two advantages as well. On one hand, it is easier to fill the gap with bone chips than to cut a bone block precisely to a shape that fits between the shell and the residual bone. On the other hand, angiogenesis of bone chips appears to be favourable compared to a thick cortical bone block, which is a biological advantage.

Shells thinner than 1 mm provide a vertical dimension to the graft and prevent the resorption of particulate bone chips. Perforations in the cortical plate of the residual bone allow blood supply and vascularization of the bone chips. The modified shell technique also has a major impact on the long-term stability of an implant-supported prosthesis. Using only one donor site could help to reduce postoperative morbidity and complication rates. Further, the described vertical shell technique offers the possibility of vertical bone reconstruction as well as the intraoral harvesting of autologous bone for larger vertical augmentation procedures. Regeneration of vital bone then provides suitable conditions for implant placement [22].

Furthermore, fitting of autologous osseous plates to follow the anatomical curvature of the anterior mandible leads to optimal healing and superior cosmetic outcomes [30]. As an alternative to our treatment method using the modified shell technique with autologous bone plates and the GBR technique, the Kerfed-Khoury split bone block technique could have been used [31]. This is because when it comes to grafting techniques, onlay bone grafts, particularly Khoury's split bone block technique (SBBT), have shown positive results in complex alveolar bone augmentations [32]. However, this work was not published until 13 years after treatment began, and based on their knowledge, experience, and todays prosthetic possibilities, the authors would have solved this demanding case with only two implants.

Finally, we would be paramount to underline that this approach is suitable also in treatment of maxillary complex fracture, and in other maxillofacial surgery fields, such as orofacial surgery. The aim should always be to achieve the maximum aesthetic and functional for reconstruction results with such a minimally invasive technique [33–36].

In our case, the marginal bone loss after a complicated comminuted fracture following vertical augmentation using the modified shell technique could be measured both buccally and lingually with annual CBCT scans using GIMP© software. Observing whether there is a difference in bone resorption between the two augmentation techniques—bone block and GBR—may provide crucial information about which method may be more successful in supporting vertical bone augmentation.

Since the patient had no clinical or pathological symptoms, it can be assumed that the overall result so far has been a complete success. There were also no signs of morbidity, numbness, or nerve injury. Overall, the patient was very satisfied with the functional and aesthetic outcomes. This positive outcome is consistent with results from the literature, which describes success rates of surgical procedures ranging from 60% to 100% for GBR, 92% to 100% for onlay bone grafts, and 98% to 100% for ridge augmentation methods. Implant survival rates also ranged from 92% to 100% for GBR [37].

In the present study, the marginal bone loss around the implants in the buccal region ranged from 0.46 to 1.29 mm, which was greater than in the lingual region, where the marginal bone loss ranged from 0.06 to 0.97 mm during the observation period. This may suggest that supporting vertical augmentation with a membrane only is somewhat more successful in this case than using a membrane and a plate of cortical bone. However, a more appropriate assessment would be that both methods proved successful as the lingual and buccal environments are different.

Over the past decade, the use of CBCT has increased in dental practice and has become a standard radiological diagnostic method [38]. For a given indication, we need diagnostically appropriate images. This has led to an adaptation of the traditional ALARA principle towards ALADAIP (As Low as Diagnostically Acceptable being Indication-oriented and Patient-specific), as stated in the Dimitra Research Group statement. Furthermore, radiation doses should be considered indication-oriented and patient-specific. Only if the strategy of time-dependent monitoring of indication-based and patient-specific radiation doses is observed can a dentist truly adhere to the ALADIP principles for optimisation and radiation protection in daily practice [39]. To date, two-dimensional intraoral radiographs are still considered the most important tool when it comes to the postoperative monitoring of implants [40].

Considering that we need to evaluate three-dimensional bone healing, including morphologic, volumetric, and trabecular remodelling, one might wonder what can be observed and diagnosed by simply looking at the approximal peri-implant 2D bone.

The only way to fully capture the peri-implant tissue is to obtain a true 3D view of the clinical situation, which brings us back to the three-dimensional imaging of the peri-implant bone [41, 42].

In our case, the imaging was gathered from three different centres, which allowed accurate documentation. Based on the data showing minimal bone loss after a comminuted fracture and bone augmentation with the modified shell and the GBR technique, it is not indicated to perform an annual CBCT examination in the future.

## 6. Conclusion

The complication-free course after the treatment of a mandibular comminuted fracture is an important prerequisite for reconstructing the anterior ridge. The combination of the modified shell technique with autologous bone plates and the GBR technique is a reliable method to ensure new bone formation in the anterior ridge with a natural curvature.

CBCT is also an excellent method to evaluate bone resorption around implants, but based on the minimal bone resorption over this 12-year observation period, an annual CBCT examination is exaggerated.

## Data Availability

The clinical data used to support the findings of this study are included within the article.

## Abbreviations

CT: Computed tomography
CBCT: Cone beam computed tomography
GBR: Guided bone regeneration
SBBT: Split bone block technique.
